# Impact of cumulative exposure to anticholinergic and sedative drugs on cognition in older adults: a memory clinic cohort study

**DOI:** 10.1186/s13195-024-01530-8

**Published:** 2024-07-23

**Authors:** Elsa Reallon, Frédéric Gervais, Claire Moutet, Virginie Dauphinot, Pauline Desnavailles, Teddy Novais, Pierre Krolak-Salmon, Antoine Garnier-Crussard, Christelle Mouchoux, MEMORA group, Zaza Makaroff, Marie-Hélène Coste, Sophie Dautricourt, Isabelle Rouch, Keren Danaila, Aziza Waissi, Jean-Michel Dorey, Alain Sarciron, Yves Guilhermet, Sylvain Gaujard, Pierre Grosmaître, Thomas Gilbert, Julien Vernaudon, Virginie Desestret, Clémence Grangé, Frederic Gervais, Achille Teillac, Mathieu Verdurand, Floriane Delphin-Combe, Anthony Bathsavanis, Romain Bachelet, Mohamed-Nour Temedda

**Affiliations:** 1 Pharmacy Department, Charpennes Hospital, Hospices Civils de Lyon, 27 Rue Gabriel Péri, 69100 Villeurbanne, France; 2 Clinical and Research Memory Center of Lyon, Lyon Institute For Aging, Hospices Civils de Lyon, 69100 Villeurbanne, France; 3 Research on Healthcare Performance (RESHAPE), University Lyon 1, INSERM U1290, Lyon, France; 4 Eduwell Team, Lyon Neuroscience Research Center (CRNL), INSERM U1028, CNRS UMR5292, UCBL1, Lyon, France; 5 Normandie Univ, UNICAEN, INSERM, U1237, PhIND “Physiopathology and Imaging of Neurological Disorders”, NeuroPresage Team, Cyceron, 14000 Caen, France

**Keywords:** Dementia, Alzheimer’s Disease, Hypnotics and Sedatives, Anticholinergic, Potentially Inappropriate Medication

## Abstract

**Background:**

Long-term exposure to anticholinergic and sedative drugs could be a modifiable risk factor for cognitive decline. The objective of this study was to measure the association between previous cumulative anticholinergic and sedative drug exposure (Drug Burden Index) and cognitive decline.

**Methods:**

A cohort study (MEMORA cohort) was conducted in a French memory clinic for patients attending a consultation between November 2014 and December 2020, with at least 2 Mini-Mental State Examination (MMSE) measurements (≥ 6 months apart) and available medication data from the local Primary Health Insurance Fund database (*n* = 1,970). Drug Burden Index was linearly cumulated until each MMSE measurement and was used to categorise patients according to their level of exposure (no exposure, moderate, or high). The longitudinal association between Drug Burden Index and MMSE was assessed using a multivariate linear mixed model, adjusted for age, education level, anxiety disorders, depressive disorders, functional autonomy, and behavioural disorders.

**Results:**

Overall, 1,970 patients were included with a mean follow-up duration of 2.78 years (± 1.54) and 2.99 visits per patients (5,900 MMSE + Drug Burden Index measurements collected). At baseline, 68.0% of patients had moderate cumulative anticholinergic and sedative drug exposure and a mean MMSE of 21.1. MMSE decrease was steeper in patients with moderate and high Drug Burden Index ( -1.74 and -1.70/year, respectively) than in patients with no exposure (-1.26/year) after adjusting for age, education, anxiety and depressive disorders, functional autonomy, and behavioural disorders (*p* < 0.01).

**Conclusions:**

Long-term exposure to anticholinergic and sedative drugs is associated with steeper cognitive decline. Medication review focusing on de-prescribing these drugs could be implemented early to reduce cognitive impairment.

## Background

With worldwide aging, cognitive impairment has become a growing concern. The primary etiology for major neurocognitive disorders—i.e., cognitive impairment associated with autonomy loss—is Alzheimer’s disease [1], which is associated with severe consequences for functional autonomy [2, 3]. Strategies to limit cognitive decline and the global burden of Alzheimer’s disease are needed. The use of anticholinergic and sedative drugs has been associated with acute cognitive impairment and other central adverse events such as delirium and falls [4–9]. Their use constitutes a potential modifiable factor for the prevention of cognitive impairment: reducing long-term exposure to anticholinergic and sedative drugs offers the opportunity to slow cognitive decline and its consequences.

Several longitudinal studies [10–14] have assessed the long-term association between cognition and anticholinergic or sedative burden using the Drug Burden Index (DBI). This scale is considered the most appropriate tool for assessing longitudinal exposure to these drugs [15]. Although most of these studies show that anticholinergic and sedative burden seem to negatively impact cognition [10–13], none of them considered the potential cumulative effect of these drugs; all of these studies measured the DBI cross-sectionally, i.e., at the same time as the assessment of cognitive status, and some only considered a single DBI measurement [11, 12].

However, the impact of anticholinergic and sedative drugs on cognition is likely to be due to previous exposure and may depend on the amount and duration of this exposure. To address this issue, it would thus seem relevant to evaluate the cumulative exposure to anticholinergic and sedative drugs years before cognition assessment as well as the change in cognition over time according to therapeutic changes.

## Methods

The main objective of this study was to measure the association between previous cumulative exposure to anticholinergic and sedative drugs and cognition in a longitudinal real-life cohort.

### Study setting, design, and participants

MEMORA is a multicentre prospective cohort study conducted throughout the patient's care pathway in Memory clinics of Lyon, France. MEMORA aims to investigate factors associated with changes in functional autonomy, cognitive performance, and Behavioral and Psychological Symptoms in Dementia (BPSD) over time in individuals receiving routine care [16]. MEMORA includes every patient who underwent a consultation at a memory clinic for a cognitive complaint, from November 2014.

The data of participants in the present study were extracted from a 6-year sample of MEMORA patients (2014–2020). Patients whose clinical data and medication data from the local branch of the Primary Health Insurance Fund (PHIF) database were available were included. Patients with missing data regarding one of these two criteria were excluded. This study followed the STROBE checklist from the EQUATOR guidelines [17].

### Anticholinergic and sedative exposure

The level of exposure to anticholinergic and sedative drugs for each patient was measured using the Drug Burden Index (DBI) [18], which was developed for older people based on pharmacological principles. The DBI is a daily score and is calculated according to the following formula:

$$DBI={DBI}_{AC}+{DBI}_{S}= \sum_{i=1}^{{n}_{d}}\frac{{D}_{i(AC)}}{{\delta }_{i\left(AC\right)}+{D}_{i\left(AC\right)} }+ \sum_{i=1}^{{n}_{d}}\frac{{D}_{i(S)}}{{\delta }_{i\left(S\right)}+{D}_{i\left(S\right)}}$$

where ${D}_{i}$ represents the daily dose of medication $i$ ($i$ = 1, …, ${n}_{d}$) with anticholinergic and/or sedative proprieties and ${\delta }_{i}$ represents the minimal effective dose of this medication according to the World Health Organization (WHO) Defined Daily Dose [19]. In the DBI calculation originally developed by Hilmer et al. [18], ${\delta }_{i}$ represents the recommended minimum daily dose approved by the U.S. Food and Drug Administration (FDA). To enable the comparison of DBI across countries, a previous study demonstrated the equivalence between the two DBI calculation formulae [20]. The list of medications with anticholinergic or sedative properties was obtained from the literature and adapted according to French practices [20–22].

**Fig. 1:**
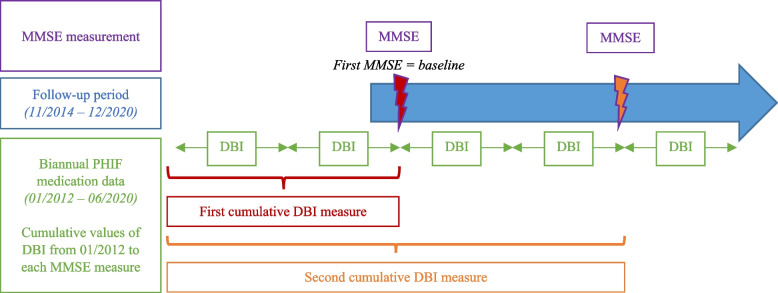
Timeline description of the study. DBI: Drug Burden Index; MMSE: Mini-Mental State Examination; PHIF: Primary Health Insurance Fund

The medications received by the included patients was collected through a PHIF extraction, where all prescribed and reimbursed drugs are registered when dispensed. For each patient included, medication data were available from 2 years before the first memory consultation until the last one. PHIF data included the name, dosage and quantity of drugs dispensed, combined by semester. To calculate the daily DBI, a mean daily dose for all anticholinergic and sedative drugs was derived from the 6-month drug consumption. The cumulative DBI was computed by adding the daily DBI over the entire available period prior to each cognitive assessment (see Fig. 1).

Patients were then divided into 3 groups based on the DBI’s standard daily classification: no exposure to anticholinergic and sedative drugs (DBI = 0), moderate exposure (0 > DBI > 1), high exposure (DBI ≥ 1). The usual daily thresholds were multiplied by the number of medication follow-up days available before each Mini-Mental State Examination (MMSE) measurement.

### Mini-Mental State Examination (MMSE)

Comprehensive cognitive assessment was performed by a physician, a nurse, or a psychologist at baseline and at each consultation. Cognitive impairment was assessed using the standardized Mini-Mental State Examination (MMSE) [23] and range from 0 (severe cognitive impairment) to 30 (no impairment). A minimum of two MMSE measures separated by at least 6 months had to be available to include a patient in the study. In the following analysis, the term “baseline” refers to the first MMSE measurement of the patient.

### Covariates

Baseline characteristics, such as age, sex, educational level, functional autonomy level, and BPSD, were collected. Functional autonomy was assessed by the 8-item, version of the Lawton Instrumental Activities of Daily Living (IADL) score [24], with a higher score indicating greater functional autonomy. BPSD was measured using the Neuropsychiatric Inventory (NPI) score [25], which ranges from 0 to 144; a higher score indicates a greater number/severity of disorders. Anxiety and depressive disorders were collected only when they were suspected as etiological diagnoses for the cognitive complaint.

### Statistical analysis

The participants’ characteristics are presented as numbers and percentages for qualitative variables and means and standard deviations (SD) for quantitative variables. Baseline characteristics were compared among the 3 groups at the anticholinergic and sedative exposure levels using the chi-squared test for categorical variables, and analysis of variance (ANOVA) for continuous variables.

A multivariable linear mixed model with a random intercept and slope was built to examine the longitudinal relationships between anticholinergic and sedative burden and cognitive function. This model allows time-series to vary between individuals and was adjusted for the baseline covariates age, educational level, anxiety disorders, depressive disorders, IADL, and NPI. The duration (in days) of the medication follow-up data available before each MMSE and DBI measurement was considered a time-dependent variable in the model. This model will produce an estimation of MMSE variation (stated as estimate and *p*-value) according to each outcomes tested in the analyses. The results will also present the interaction between natural MMSE variation during the follow-up length and anticholinergic and sedative burden.

Missing values were imputed only for covariates in the multivariate analysis, with Multiple Imputation by Chained Equations (MICE) methods.

Descriptive analyses were performed with SPSS Statistics for Windows (v21.0; IBM). The linear mixed model was performed in R Statistical Software (v4.1.3; R Core Team 2022) [26]. All tests were two tailed, and a priori *p* value less than 0.05 was considered to indicate statistical significance.

## Results

### Population selection

**Fig. 2:**
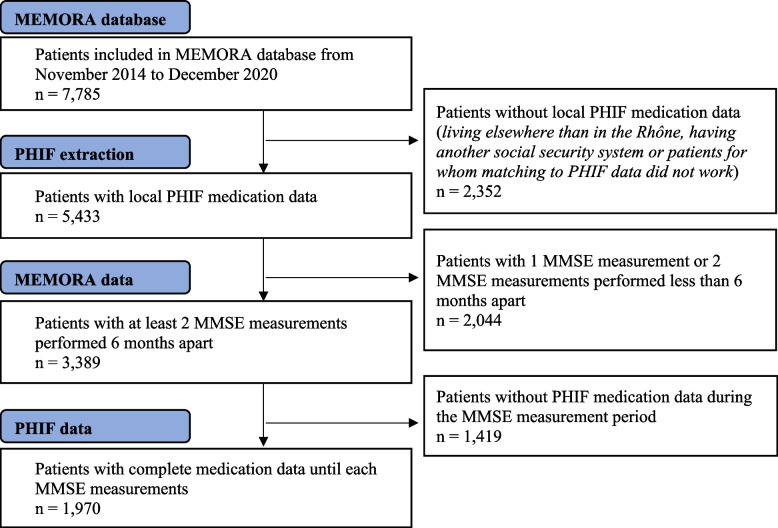
Inclusion flow-chart. MMSE: Mini-Mental State Examination; PHIF: Primary Health Insurance Fund

In total, between November 2014 and December 2020, 1,970 patients were included in the analysis (Fig. 2). Among these, 5,900 MMSE and DBI scores were collected, corresponding to a mean of 2.99 measurements per patient (range 2–10). The mean medication follow-up (PHIF data) length prior to each MMSE measurement was 2.78 ± 1.54 years.

### Characteristics of the population

The included population included a majority of women (60.7%), with a secondary level of education (34.9%) and a mean (SD) age of 79.6 (± 7.3) years. At baseline, the mean MMSE score was 21.1 (± 5.7), 15.9% of patients had no anticholinergic or sedative exposure, 68.0% had moderate exposure, and 16.1% had high exposure (Table 1). Anxiety and depressive disorders were involved in the etiology of cognitive complaints in less than 3% of the population (2.0% and 2.9%, respectively).

**Table 1:** Baseline population characteristics according to the baseline level of exposure to anticholinergic and sedative drugs

Baseline DBI level:	No exposure *n* = 314	Moderate exposure *n* = 1339	High exposure *n* = 317		Total *n* = 1970
	n (%) or mean ± sd			*p*-value	n (%) or mean ± sd
Sex					
Female	179 (57.0)	814 (60.8)	202 (63.7)	0.222	1195 (60.7)
Age (years)	79.8 ± 7.9	79.8 ± 7.1	78.8 ± 7.4	0.076	79.6 ± 7.3
Educational level *n* = 1,862				0.001	
None	46 (15.3)	191 (15.1)	68 (22.8)		305 (15.5)
Primary	79 (26.2)	414 (32.8)	94 (31.5)		587 (29.8)
Secondary and further	176 (58.5)	658 (52.1)	136 (45.6)		970 (49.3)
IADL (/8) *n* = 1,927	4.8 ± 2.3	4.5 ± 2.4	4.0 ± 2.4	< 0.001	4.5 ± 2.4
NPI (/144) *n* = 1,522	16.2 ± 15.0	19.9 ± 16.1	23.8 ± 19.1	< 0.001	20.0 ± 16.6
Anxiety disorders *n* = 1,887	5 (1.7)	27 (2.1)	7 (2.3)	0.851	39 (2.0)
Depressive disorders *n* = 1,887	4 (1.3)	32 (2.5)	21 (7.0)	< 0.001	57 (2.9)
MMSE (/30)	21.6 ± 5.5	21.1 ± 5.6	20.5 ± 5.9	0.037	21.1 ± 5.7

*DBI* Drug Burden Index, *IADL* Instrumental Activities of Daily Living score, *MMSE* Mini Mental State Examination, *NPI *Neuropsychiatric Inventory score

In the included population, the last available suspected aetiologic diagnosis for cognitive complaints was mainly Alzheimer’s disease (59.1%), followed by vascular dementia (12.1%) and dementia with Lewy bodies (3.4%). The aetiologic diagnosis was unknown for 12.4% of the patients. At baseline, educational level, functional autonomy (IADL), and MMSE scores were significantly higher in patients without exposure to anticholinergic and sedative drugs, and they also were significantly less affected by behavioural (NPI) or depressive disorders than patients with moderate or high exposure.

### Multivariate linear mixed model (random intercept and slope)

Significant associations with MMSE scores were found for moderate exposure to anticholinergic and sedative drugs (β = 0.61, *p* value = 0.049), follow-up duration (β = -1.26, *p* value < 0.001), depressive disorders (β = 1.94, *p* value = 0.001), educational level (with an increasing effect), and functional autonomy (β = 0.89, *p* value < 0.001; Table 2).

**Table 2:** Multivariate linear mixed model with MMSE score as the dependent variable

Parameters	Estimate	Test (df)	*p* value
DBI			
No exposure	Ref	-	-
Moderate exposure	0.61	1.97 (2839.00)	0.049
High exposure	0.34	0.80 (568.89)	0.423
Age	-0.01	-0.86 (5737.85)	0.388
Follow-up length (years)	-1.26	-9.25 (5000.95)	< 0.001
Anxiety disorders	0.94	1.07 (39.56)	0.290
Depressive disorders	1.94	3.23 (211.88)	0.001
Educational level			
Secondary and further	Ref	-	-
Primary	-1.95	-7.87 (138.96)	< 0.001
None	-4.13	-13.58 (259.15)	< 0.001
IADL	0.89	18.87 (766.47)	< 0.001
NPI	-0.01	-1.41 (71.19)	0.163
Follow-up length x DBI			
No exposure	Ref	-	-
Moderate exposure	-0.48	-2.84 (2005.59)	0.005
High exposure	-0.44	-3.51 (4759.00)	< 0.001

*DBI* Drug Burden Index, *df* degree of freedom, *IADL* Instrumental Activities of Daily Living, *NPI* Neuropsychiatric Inventory

A cognitive decline of 1.26 points per year on the MMSE (β = -1.26,* p* value < 0.001) was observed for patients without any anticholinergic or sedative exposure. With moderate exposure to these drugs extent of cognitive decline increased by 0.48 points per year (*p* value < 0.001), and extent of cognitive decline increased by 0.48 points per year (*p* value < 0.001), and extent of cognitive decline increased by 0.44 points per year with high exposure (*p* value = 0.005, Table 2). Overall, the MMSE score significantly decreased by 1.74 points per year for patients with moderate DBI scores ((-1.26) + (-0.48) = (-1.74)) and 1.70 points per year for patients with high DBI scores ((-1.26) + (-0.44) = (-1.70).

## **Discussion**

The present longitudinal study showed that moderate and high cumulative long-term exposure to anticholinergic and sedative drugs in older adults was associated with an additional decrease in MMSE score of 0.48 and 0.44 points per year, respectively, further strengthening the evidence that anticholinergic and sedative drug exposure negatively impacts cognition in older adults.

The main finding of the present study is consistent with previous results from both longitudinal and cross-sectional studies [10–12, 18, 27–31]. However, the present study is the first, to our knowledge, to estimate cumulative drug exposure several years before cognitive assessment, providing stronger evidence of the negative impact of anticholinergic and sedative drugs on cognition. These findings provide evidence that the impact of medication on cognition should be considered based not only on single daily exposure (as measured by the DBI daily score) but also on cumulative exposure over time.

The present results show no trend towards a dose‒response effect between moderate and high exposure to anticholinergic and sedative drugs. This could be explained by the high proportion of patients in the moderate-exposure group, a proportion nearly twice as high as the 20-35% of patients in the moderate-exposure group previously reported [13, 32–34]. It is possible that the extrapolation of the daily DBI to a cumulative DBI using the proposed approach led to miscategorisation of patients. Further methodological research, such as cluster analysis, will be carried out to better delineate the cumulative exposure groups that could characterize patients in these longitudinal studies.

However, several studies in the literature have shown no association between anticholinergic or sedative exposure and cognition [13, 14, 35–37]. These discordant results may be explained by the heterogeneity in the tools used due to the high number of validated scales available to measure cognition and drug exposure. The DBI itself, which was used herein because it is described as the most suitable measure for longitudinal studies [15], also has limitations. First, it does not take into account the different anticholinergic levels of drugs. Second, it represents a daily burden, and thresholds do not exist for categorising long-term exposure levels to anticholinergic and sedative drugs. Finally, different results might be produced for a single patient depending on the country or the authors (i.e., the minimal effective dose in the DBI formula is calculated according to national references, and the drug lists used can vary from one author to another) [38–42].

Moreover, most studies did not control for confounding factors such as behavioural disorders and functional autonomy loss [10–14, 26, 33]. On the one hand, these factors are commonly associated with poorer cognition, and on the other hand, patients with these symptoms are more likely to receive anticholinergic or sedative drugs. These potential confounding factors, such as the NPI and IADL scores, were included in the present multivariate model.

The use of the PHIF to collect data might represent the main limitation of the present study. Due to its nature, medication data can only be obtained biannually and thus do not reflect the true daily dose needed to calculate DBI. Moreover, although data obtained from the PHIF allow treatment compliance to be ensured, as the PHIF presents drugs actually purchased by patients in pharmacies, it does not consider nonreimbursed or over-the-counter drugs. However, we assume that this would not impact the exposure group distribution as very few over-the-counter drugs have strong anticholinergic and sedative properties and their use is generally occasional and limited in time. More importantly, PHIF data are reliable for longitudinal studies because they reflect all medication changes during a studied period.

Exposure to medication is a modifiable risk factor that can change over time. Since 2014, deprescribing of these drugs has been a growing topic with several randomised controlled trials implemented all over the world. Anticholinergic and sedative drugs have been associated with multiple negative health outcomes [11, 15] and the main hypothesis of these trials was that stopping them would lead to improved health condition. Unexpectedly, few studies have been able to show an efficacy of their intervention to successfully deprescribe these drugs, and even fewer have been able to show an efficacy on clinical outcomes [43, 44]. To explain the mitigate results, systemic reviews and meta-analysis suspect a lack of statistical power, a too short patient follow-up time, a lack of patient support through the deprescribing process, and a lack of professional training [43, 44]. Therefore, to address the last two issues, a successful deprescribing process should involve and support patient throughout the process and bring interdisciplinary through the medication reviews process, where pharmacists and physicians may combine their medication and clinical evaluation to reach sustainable deprescribing.This process should be conducted as early as possible, preferably before the occurrence of symptoms of cognitive decline (memory complaints, falls), after which recovery is rarely complete.

Our results suggest that reducing exposure to anticholinergic and sedative drugs can slow cognitive decline (0.44 points of MMSE per 12 months). This effect on cognitive function is similar to recent results on the efficacy of disease-modifying therapies such as anti-amyloid immunotherapies that have shown a nonsignificant improvement in MMSE score of 0.3 points per 18 months [45]. Deprescribing anticholinergic and sedative drugs combined with disease-modifying therapies could be an effective holistic care pathway for slowing cognitive decline, to be confirmed by interventional studies.

## Conclusions

The findings reported herein show that long-term anticholinergic and sedative exposure was significantly associated with cognitive decline. The effect of this cumulative exposure must be further explored, and additional interventional trials should investigate the benefits of stopping anticholinergic or sedative drug prescriptions whenever possible through collaborative medication review, for example. Finally, since medication exposure appears to be a modifiable risk factor for cognitive decline, prevention strategies aiming to limit the prescription of these drugs as early as possible should be considered.

## Data Availability

Study data are available on reasonable quest from the corresponding author.

## Abbreviations

BPSD: Behavioral and Psychological Symptoms in Dementia
DBI: Drug Burden Index
FDA: Food and Drug Administration
IADL: Instrumental Activities of Daily Living
MICE: Multiple Imputation by Chained Equations
MMSE: Mini-Mental State Examination
NPI: Neuropsychiatric Inventory
PHIF: Primary Health Insurance Fund
WHO: World Health Organization
