# Correction: Impact of cumulative exposure to anticholinergic and sedative drugs on cognition in older adults: a memory clinic cohort study

**DOI:** 10.1186/s13195-024-01560-2

**Published:** 2024-08-29

**Authors:** Elsa Reallon, Frédéric Gervais, Claire Moutet, Virginie Dauphinot, Pauline Desnavailles, Teddy Novais, Pierre Krolak‑Salmon, Antoine Garnier‑Crussard, Christelle Mouchoux, MEMORA group, Antoine Garnier-Crussard, Virginie Dauphinot, Zaza Makaroff, Marie-Hélène Coste, Sophie Dautricourt, Isabelle Rouch, Keren Danaila, Aziza Waissi, Jean-Michel Dorey, Alain Sarciron, Yves Guilhermet, Sylvain Gaujard, Pierre Grosmaître, Thomas Gilbert, Julien Vernaudon, Virginie Desestret, Clémence Grangé, Christelle Mouchoux, Frederic Gervais, Achille Teillac, Claire Moutet, Mathieu Verdurand, Floriane Delphin-Combe, Anthony Bathsavanis, Romain Bachelet, Mohamed-Nour Temedda, Pierre Krolak-Salmon

**Affiliations:** 1 Pharmacy Department, Charpennes Hospital, Hospices Civils de Lyon, 27 Rue Gabriel Péri, 69100 Villeurbanne, France; 2 Clinical and Research Memory Center of Lyon, Lyon Institute For Aging, Hospices Civils de Lyon, 69100 Villeurbanne, France; 3 Research On Healthcare Performance (RESHAPE), University Lyon 1, INSERM U1290, Lyon, France; 4 Eduwell Team, Lyon Neuroscience Research Center (CRNL), INSERM U1028, CNRS UMR5292, UCBL1, Lyon, France; 5 Normandie Univ, UNICAEN, INSERM, U1237, PhIND “Physiopathology and Imaging of Neurological Disorders”, NeuroPresage Team, Cyceron, 14000 Caen, France

**Correction: Alz Res Therapy 16, 163 (2024)**

**https://doi.org/10.1186/s13195-024-01530-8**

Following the publication of the original article [1], seven paragraphs of the Discussion section and the last sentence of the second paragraph under “Multivariate linear mixed model (random intercept and slope)” section were unintentionally omitted from this article during Typesetting process.

The last paragraph of “Multivariate linear mixed model (random intercept and slope)” section should have read:

A cognitive decline of 1.26 points per year on the MMSE (β = -1.26, *p* value < 0.001) was observed for patients without any anticholinergic or sedative exposure. With moderate exposure to these drugs extent of cognitive decline increased by 0.48 points per year (*p* value < 0.001), and extent of cognitive decline increased by 0.48 points per year (*p* value < 0.001), and extent of cognitive decline increased by 0.44 points per year with high exposure (*p* value = 0.005, Table 2). Overall, the MMSE score significantly decreased by 1.74 points per year for patients with moderate DBI scores ((-1.26) + (-0.48) = (-1.74)) and 1.70 points per year for patients with high DBI scores ((-1.26) + (-0.44) = (-1.70).

The Discussion section should have read:

**Discussion**

The present longitudinal study showed that moderate and high cumulative long-term exposure to anticholinergic and sedative drugs in older adults was associated with an additional decrease in MMSE score of 0.48 and 0.44 points per year, respectively, further strengthening the evidence that anticholinergic and sedative drug exposure negatively impacts cognition in older adults.

The main finding of the present study is consistent with previous results from both longitudinal and cross-sectional studies [10–12,18,27–31]. However, the present study is the first, to our knowledge, to estimate cumulative drug exposure several years before cognitive assessment, providing stronger evidence of the negative impact of anticholinergic and sedative drugs on cognition. These findings provide evidence that the impact of medication on cognition should be considered based not only on single daily exposure (as measured by the DBI daily score) but also on cumulative exposure over time.

The present results show no trend towards a dose‒response effect between moderate and high exposure to anticholinergic and sedative drugs. This could be explained by the high proportion of patients in the moderate-exposure group, a proportion nearly twice as high as the 20–35% of patients in the moderate-exposure group previously reported [13,32–34]. It is possible that the extrapolation of the daily DBI to a cumulative DBI using the proposed approach led to miscategorisation of patients. Further methodological research, such as cluster analysis, will be carried out to better delineate the cumulative exposure groups that could characterize patients in these longitudinal studies.

However, several studies in the literature have shown no association between anticholinergic or sedative exposure and cognition [13,14,35–37]. These discordant results may be explained by the heterogeneity in the tools used due to the high number of validated scales available to measure cognition and drug exposure. The DBI itself, which was used herein because it is described as the most suitable measure for longitudinal studies [15], also has limitations. First, it does not take into account the different anticholinergic levels of drugs. Second, it represents a daily burden, and thresholds do not exist for categorising long-term exposure levels to anticholinergic and sedative drugs. Finally, different results might be produced for a single patient depending on the country or the authors (i.e., the minimal effective dose in the DBI formula is calculated according to national references, and the drug lists used can vary from one author to another) [38–42].

Moreover, most studies did not control for confounding factors such as behavioural disorders and functional autonomy loss [10–14,26,33]. On the one hand, these factors are commonly associated with poorer cognition, and on the other hand, patients with these symptoms are more likely to receive anticholinergic or sedative drugs. These potential confounding factors, such as the NPI and IADL scores, were included in the present multivariate model.

The use of the PHIF to collect data might represent the main limitation of the present study. Due to its nature, medication data can only be obtained biannually and thus do not reflect the true daily dose needed to calculate DBI. Moreover, although data obtained from the PHIF allow treatment compliance to be ensured, as the PHIF presents drugs actually purchased by patients in pharmacies, it does not consider nonreimbursed or over-the-counter drugs. However, we assume that this would not impact the exposure group distribution as very few over-the-counter drugs have strong anticholinergic and sedative properties and their use is generally occasional and limited in time. More importantly, PHIF data are reliable for longitudinal studies because they reflect all medication changes during a studied period.

Moreover, below are the references details of the citations mentioned from these paragraphs.

27. Bierman EJM, Comijs HC, Gundy CM, Sonnenberg C, Jonker C, Beekman ATF. The effect of chronic benzodiazepine use on cognitive functioning in older persons: good, bad or indifferent? Int J Geriatr Psychiatry. 2007;22(12):1194‑200.

28. Cao YJ, Mager DE, Simonsick EM, Hilmer SN, Ling SM, Windham BG, et al. Physical and cognitive performance and burden of anticholinergics, sedatives, and ACE inhibitors in older women. Clin Pharmacol Ther. 2008;83(3):422‑9.

29. Lechevallier-Michel N, Molimard M, Dartigues JF, Fabrigoule C, Fourrier-Réglat A. Drugs with anticholinergic properties and cognitive performance in the elderly: results from the PAQUID Study. Br J Clin Pharmacol. 2005;59(2):143‑51.

30. Nebes RD, Pollock BG, Meltzer CC, Saxton JA, Houck PR, Halligan EM, et al. Serum anticholinergic activity, white matter hyperintensities, and cognitive performance. Neurology. 2005;65(9):1487‑9.

31. Uusvaara J, Pitkala KH, Kautiainen H, Tilvis RS, Strandberg TE. Detailed cognitive function and use of drugs with anticholinergic properties in older people: a community-based cross-sectional study. Drugs Aging. 2013;30(3):177‑82.

32. Espaulella-Ferrer M, Molist-Brunet N, Espaulella-Panicot J, Sevilla-Sánchez D, Puigoriol-Juvanteny E, Otero-Viñas M. Medication Assessment in an Older Population during Acute Care Hospitalization and Its Effect on the Anticholinergic Burden: A Prospective Cohort Study. Int J Environ Res Public Health. 2023;20(7):5322.

33. Katzenberger B, Koller D, Strobl R, Kisch R, Sanftenberg L, Voigt K, et al. Exposure to anticholinergic and sedative medication is associated with impaired functioning in older people with vertigo, dizziness and balance disorders-Results from the longitudinal multicenter study MobilE-TRA. Front Pharmacol. 2023;14:1136757.

34. Gnjidic D, Le Couteur DG, Abernethy DR, Hilmer SN. Drug burden index and beers criteria: impact on functional outcomes in older people living in self-care retirement villages. J Clin Pharmacol. 2012;52(2):258‑65.

35. Ancelin ML, Artero S, Portet F, Dupuy AM, Touchon J, Ritchie K. Non-degenerative mild cognitive impairment in elderly people and use of anticholinergic drugs: longitudinal cohort study. BMJ. 2006;332(7539):455‑9.

36. Fox C, Livingston G, Maidment ID, Coulton S, Smithard DG, Boustani M, et al. The impact of anticholinergic burden in Alzheimer’s dementia-the LASER-AD study. Age Ageing. 2011;40(6):730‑5.

37. Whalley LJ, Sharma S, Fox HC, Murray AD, Staff RT, Duthie AC, et al. Anticholinergic drugs in late life: adverse effects on cognition but not on progress to dementia. J Alzheimers Dis. 2012;30(2):253‑61.

38. By the 2019 American Geriatrics Society Beers Criteria® Update Expert Panel. American Geriatrics Society 2019 Updated AGS Beers Criteria® for Potentially Inappropriate Medication Use in Older Adults. J Am Geriatr Soc. 2019;67(4):674‑94.

39. O’Mahony D, O’Sullivan D, Byrne S, O’Connor MN, Ryan C, Gallagher P. STOPP/START criteria for potentially inappropriate prescribing in older people: version 2. Age Ageing. 2015;44(2):213‑8.

40. Laroche ML, Bouthier F, Merle L, Charmes JP. [Potentially inappropriate medications in the elderly: a list adapted to French medical practice]. Rev Med Interne. 2009;30(7):592‑601.

41. Renom-Guiteras A, Meyer G, Thürmann PA. The EU(7)-PIM list: a list of potentially inappropriate medications for older people consented by experts from seven European countries. Eur J Clin Pharmacol. 2015;71(7):861‑75.

42. Roux B, Berthou-Contreras J, Beuscart JB, Charenton-Blavignac M, Doucet J, Fournier JP, et al. REview of potentially inappropriate MEDIcation pr[e]scribing in Seniors (REMEDI[e]S): French implicit and explicit criteria. Eur J Clin Pharmacol. 2021;77(11):1713‑24.

The original article [1] has been updated. Note: References were renumbered accordingly in ascending numerical order in the original article.
